# British laypeople’s attitudes towards gradual sedation, sedation to unconsciousness and euthanasia at the end of life

**DOI:** 10.1371/journal.pone.0247193

**Published:** 2021-03-26

**Authors:** Antony Takla, Julian Savulescu, Andreas Kappes, Dominic J. C. Wilkinson

**Affiliations:** 1 Faculty of Medicine, Nursing and Health Science, Monash University, Clayton, Victoria, Australia; 2 Oxford Uehiro Centre for Practical Ethics, Faculty of Philosophy, University of Oxford, Oxford, United Kingdom; 3 Murdoch Children’s Research Institute, Melbourne, Australia; 4 School of Arts and Social Sciences, Department of Psychology, City University of London, London, United Kingdom; 5 John Radcliffe Hospital, Oxford, United Kingdom; Rowan University School of Osteopathic Medicine, UNITED STATES

## Abstract

**Background:**

Many patients at the end of life require analgesia to relieve pain. Additionally, up to 1/5 of patients in the UK receive sedation for refractory symptoms at the end of life. The use of sedation in end-of-life care (EOLC) remains controversial. While gradual sedation to alleviate intractable suffering is generally accepted, there is more opposition towards deliberate and rapid sedation to unconsciousness (so-called “terminal anaesthesia”, TA). However, the general public’s views about sedation in EOLC are not known. We sought to investigate the general public’s views to inform policy and practice in the UK.

**Methods:**

We performed two anonymous online surveys of members of the UK public, sampled to be representative for key demographic characteristics (n = 509). Participants were given a scenario of a hypothetical terminally ill patient with one week of life left. We sought views on the acceptability of providing titrated analgesia, gradual sedation, terminal anaesthesia, and euthanasia. We asked participants about the intentions of doctors, what risks of sedation would be acceptable, and the equivalence of terminal anaesthesia and euthanasia.

**Findings:**

Of the 509 total participants, 84% and 72% indicated that it is permissible to offer titrated analgesia and gradual sedation (respectively); 75% believed it is ethical to offer TA. Eighty-eight percent of participants indicated that they would like to have the option of TA available in their EOLC (compared with 79% for euthanasia); 64% indicated that they would potentially wish for TA at the end of life (52% for euthanasia). Two-thirds indicated that doctors should be allowed to make a dying patient completely unconscious. More than 50% of participants believed that TA and euthanasia were non-equivalent; a third believed they were.

**Interpretation:**

These novel findings demonstrate substantial support from the UK general public for the use of sedation and TA in EOLC. More discussion is needed about the range of options that should be offered for dying patients.

## Introduction

Pain is one of the most common symptoms experienced at the end of life; some studies report that up to 84% of patients require opioids in their final week before death [1, 2]. Their use has often been justified by physicians and ethicists using the doctrine of double effect, although recent evidence indicates that judicious use of analgesia and opioids does not necessarily hasten death, [1, 3–5]. In addition to standard analgesia, international estimates suggest that between 15% to 48% of patients receiving end of life care (EOLC) are given sedation in the dying phase [6, 7]. In 2010, 19% of 2,923 UK physicians attending a dying patient reported the use of continuous deep sedation, usually in response to perceived intractable suffering [8].

There are a variety of terms used for sedation in dying patients. ‘Terminal Sedation’ (TS), Palliative Sedation (PS) or Continuous Deep Sedation (CDS) are commonly used interchangeably [9, 10]. The practice is often divided into two broad categories:

- Gradual, proportional or parsimonious sedation [4],
- Deliberate and rapid sedation to unconsciousness (or, terminal anaesthesia) [4, 11, 12].

In gradual sedation, medicines are titrated based on patient symptoms and their severity [13]. The aim is to use the lowest dose needed to alleviate suffering whilst preserving consciousness where possible [14]. Such a practice is quite widespread [6, 7]. In contrast, deliberate and rapid sedation to unconsciousness typically involves the administration of larger doses of sedatives intending to achieve unconsciousness. This form of end of life sedation is relatively rare in the US and the UK but is more common in other countries [11, 15]. We have elsewhere labelled it “terminal anaesthesia” (TA), to distinguish it from other forms of end of life sedation, and since it could involve the use of anaesthetic agents/doses [12]. Current UK guidance would not permit such practice [16]. The use of sedatives is limited to relief of specific symptoms (breathlessness, anxiety and agitation) while guidance recommends commencing at the lowest effective dose and titrating “as clinically indicated”. According to National Institute of Health and Care Effectiveness (NICE) guidelines, a physician may reach unconsciousness gradually (if lighter forms of sedation do not provide adequate relief) but should not directly and immediately aim at unconsciousness [16].

Most physicians believe that providing some sedation for intractable suffering is ethically acceptable [17–20]. However, there is more concern about deeper sedation or aiming directly at unconsciousness [19, 20]. Opposition towards sedation to unconsciousness in dying patients potentially relates to fears that deeper sedation will hasten death; some have referred to it as “slow euthanasia” [10, 21]. (Observational studies suggest that the use of increasing doses of sedatives in patients who are actively dying does not necessarily shorten life [22].) Some have proposed that sedating to unconsciousness until death is morally equivalent to euthanasia [4, 23]. Others have suggested moral and conceptual differences between the two practices [12, 24]. Euthanasia remains illegal in the UK.

It remains unclear, however, where the public stands on these issues. Does the public accept rapid sedation/anaesthesia at the end of life? How do attitudes to rapid sedation compare with attitudes to gradual sedation or to deliberate ending of life? While there have been multiple studies of public attitudes to euthanasia, to our knowledge, no public surveys in the UK or elsewhere have ever explored lay people’s intuitions and attitudes towards various forms of sedation in EOLC.

## Methods

### Participants

We performed two anonymous online surveys of samples of the UK population in July and August 2020. We recruited an initial sample via the online platform Prolific, aiming to recruit 200 participants stratified for age (18–40, >40) and gender. To replicate the initial survey and increase its generalisability, we then recruited a second sample via the separate platform Qualtrics, aiming for 300 participants representative of the UK general population for age, religion and ethnicity. The sample size allows us to estimate the true preference of the UK population with a 5% margin of error (90% confidence interval). In this second survey, we excluded participants who failed either of two attention check questions or who completed the survey in less than half the median completion time.

Participants were reimbursed based on the UK’s minimum hourly wage. All indicated their consent to participate by checking a consent box on the online survey platform, before they were allowed to proceed with the survey. Ethics approval for this study was provided by the Social Sciences and Humanities Inter-Divisional Research Ethics Committee of the University of Oxford [R69871/RE003].

### Survey content

We collected basic demographic information, including age, sex, ethnicity, the highest level of education, religion and religiosity using a 5-point Centrality of Religiosity scale (CRS) [25] (see full survey in S1 Appendix).

Participants were presented with a series of hypothetical scenarios involving a terminally ill patient who is in the dying phase (Box 1). Participants were told about different treatment options and asked to indicate whether each was ethically acceptable (5-point Likert scale from Definitely yes, to definitely no). Options were:

- Titrated Analgesia (gradually increasing analgesics in response to symptoms),
- Gradual Sedation (titrating sedatives in response to symptoms),
- Terminal Anaesthesia (rapid sedation to unconsciousness), and
- Euthanasia.

**Box 1. Survey scenarios relating to titrated analgesia and terminal anaesthesia (for full survey text see S1 Appendix).**

Mr. Thomson is a 75-year-old man with end-stage cancer. He has approximately one week of life left. Mr. Thomson is admitted to his local hospital because of intense pain. He has no family or friends to be with him.

**Option A**:

The doctor sees Mr. Thomson and offers him a low dose of painkillers that he thinks will help Mr. Thomson.

If Mr. Thomson’s pain does not go away after the small dose of pain killers, the doctor can gradually increase the dose until Mr. Thomson’s pain goes away. As the dose increases, the risk of side effects (like reduced consciousness) increases and it is slightly more likely that Mr. Thomson may die sooner than if he had not received the medications at all. Do you think this is an ethical treatment option to offer to Mr. Thomson?

**Option C**:

Mr. Thomson says that he does not want to take any risk of still being in pain with the gradual increase of sedatives and instead wants to be made completely unconscious and pain-free straight away.

The doctor offers to provide anaesthetic medicines (like those given during surgery) which will make him completely unconscious immediately. There is a small risk that this anaesthetic medicine will make Mr. Thomson die sooner than if he did not take it. Do you think this is an ethical treatment option to offer to him?

At the end of each section, participants were asked what they would choose for themselves or a family member if they were in the same position as the hypothetical patient and whether they would like the option available at the end of their own life.

Survey questions also investigated participants’ views about risks associated with treatment options such as possible hastening of death. Participants were asked about the stage of the patient’s dying process at which it may be ethical to offer terminal anaesthesia (i.e. expected to die <1 day, <1week, <1 month, any time, or never).

They were asked whether they viewed an unconscious patient (who remains unconscious until death) as the same as being dead, and whether sedating someone to unconsciousness until death and euthanasia are equivalent.

### Additional measurements for replication survey

For the second survey, additional questions related to the use of artificial nutrition and hydration (ANH) and the acceptability of sedation for terminally ill patients who refuse to eat and drink, (also known as voluntary palliated starvation) [26]. We presented participants with two patients who are terminally ill and who had stopped eating and drinking; one had four weeks of life left, and the second had one week of life left. Both requested to be sedated to unconsciousness until they die. Participants were asked if the doctor should administer ANH to those two patients.

### Statistical analysis

For analysis, affirmative and negative “Definitely”, and “Probably” responses were combined. Religiosity analysis was done in two ways:

- Dichotomised into “not religious” (CRS of 1.0–2.0) and “religious” (CRS of 2.1–5.1) [25] to generate odds ratios for different responses.
- Continuous spectrum (CRS of 1.0 to 5.0) to test whether increasing religiosity influenced responses.

Analysis of the data was performed using IBM SPSS Statistics version 25.0 for Mac and GraphPad Prism version 8 for Mac. We used descriptive statistics to measure the frequency of various responses. Independent t-tests and chi-square tests were used to compare the answers of different groups of participants (e.g., religious versus non-religious participants). The null hypothesis was rejected at p<0.05.

We analysed the results of the two surveys separately and combined. Since the results were very similar for most questions, we present pooled results below. (Complete analysis of the separate surveys is detailed in S2 Appendix).

## Results

Of the 212 respondents who attempted the first survey, nine did not complete it fully, and one did not provide consent, yielding 202 complete responses. For the second survey, 885 participants were screened for eligibility. Two hundred ninety-one gave incomplete answers, 282 failed at least one of two instructional manipulation checks, and five were excluded for completing it in less than half the median completion time, leaving 307 valid responses.

Five hundred and nine responses were analysed. The two survey populations were similar in ethnicity, education, and marital status. The second survey had a larger proportion of female respondents, a higher proportion of parents, were slightly older, and were more religious (Table 1).

**Table 1:** Demographic characteristics of participants.

		Survey 1 n = 202	Survey 2 n = 307	Combined n = 509
**Age**	Mean ± SD, range	37.2 ± 13.2, 18–74	44.0 ± 15.4, 18–87	41.3 ±14.9, 18–87
**Sex**	Male	101 (50%)	93 (30.3%)	194 (38.1%)
	Female	100 (49.5%)	213 (69.4%)	313 (61.4%)
	Prefer not to say	1 (0.5%)	1 (0.3%)	2 (0.5%)
**Ethnicity**	White British	173 (85.6%)	261 (85.0%)	434 (85.3%)
	Other	18 (8.9%)	10 (3.3%)	28 (5.5%)
	Middle Eastern British	3 (1.5%)	3 (1.0%)	6 (1.2%)
	Black British	3 (1.5%)	10 (3.3%)	13 (2.5%)
	Prefer not to tell	3 (1.5%)	1 (0.3%)	4 (0.8%)
	Asian British	2 (1%)	22 (7.1%)	24 (4.7%)
**Highest level of education**	Higher Education	114 (56.4%)	174 (56.7%)	288 (56.6%)
	A Levels, vocational level 3	47 (23.3%)	58 (18.9%)	105 (20.6%)
	O Level, vocational level 2	26 (12.9%)	55 (17.9%)	81 (15.9%)
	Qualification at level 1 and below	12 (5.9%)	11 (3.6%)	23 (4.5%)
	No qualification	3 (1.5%)	9 (2.9%)	12 (2.4%)
**Marital Status**	Married	110 (54.5%)	178 (58.0%)	288 (56.6%)
	Single	76 (37.6)	96 (31.3%)	172 (33.8%)
	Other (divorced, widowed, separated)	16 (7.9%)	33 (10.7%)	49 (9.6%)
**Parental Status**	Parent (1 or more children)	81 (40.1%)	173 (56.3%)	254 (50%)
	Not a parent (no children)	121 (59.9%)	134 (43.7%)	255 (50%)
**Validated Centrality of Religiosity Scale (CRS)**	Mean ± SD	2.2 ± 0.97	2.6 ± 1.1	2.4 ± 1.1
	Not religious (CRS: 1.0–2.0)	124 (61.4%)	120 (39.1%)	244 (47.9%)
	Religious (CRS: 2.1–3.9)	59 (29.2%)	136 (44.3%)	195 (38.3%)
	Very Religious (CRS: 4.0–5.0)	19 (9.4%)	51 (16.6%)	70 (13.8%)
**Religion**	Buddhist	4 (2%)	2 (0.7%)	6 (1.2%)
	Christian	89 (44%)	185 (60.2%)	274 (53.8%)
	Hindu	0 (0.0%)	2 (0.7%)	2 (0.4%)
	Jewish	1 (0.5%)	2 (0.7%)	3 (0.6%)
	Muslim	1 (0.5%)	13 (4.2%)	14 (2.8%)
	Spiritual/ Other	1 (0.5%)	21 (6.8%)	22 (4.3%)
	Not religious (atheist, agnostic, no religion)	106 (52.5%)	82 (26.7%)	188 (36.9%)

### Acceptance of various end of life care practices

For all of the end of life options, more than 70% of participants judged them ethically acceptable (Table 2). The highest proportion of respondents endorsed titrated analgesia (84%), followed by euthanasia (76%), terminal anaesthesia (75%) and gradual sedation (72%). A large proportion of respondents indicated that they would choose gradual sedation (80%) for themselves or a family member at the end of life. A smaller proportion indicated that they would choose terminal anaesthesia (65%) or euthanasia (52%). However, a large proportion (88% for TA and 79% for euthanasia) indicated that they would like these to be available as an option at the end of life.

**Table 2:** Acceptance rates of different end of life care options (n = 509).

		Ethical to offer patients.	The participant would choose it for themselves or family member.	The participant would like to have the option available to them at the end of life
**Titrated Analgesia**	Yes	83.7%	86.0%	Not assessed*
	Uncertain	11.4%	6.9%	
	No	4.9%	7.1%	
**Gradual Sedation**	Yes	72.3%	80.2%	Not assessed*
	Uncertain	15.5%	9.9%	
	No	12.2%	9.8%	
**Terminal Anaesthesia**	Yes	74.5%	64.6%	88.4%
	Uncertain	14.7%	16.5%	6.3%
	No	10.8%	18.9%	5.3%
**Euthanasia**	Yes	75.8%	51.5%	79.0%
	Uncertain	8.1%	24.6%	6.7%
	No	16.1%	23.9%	14.3%

* Participants were not asked this because this practice is already an available option for intractable suffering.

Most participants (87.8%) indicated that it is acceptable for a doctor to provide a patient with pain killers if the doctor’s intention is to relieve pain and not hasten death. This contrasts with 40% of participants who indicated that it is still acceptable to give patient’s pain killers even if the doctor’s intention is to hasten death.

**Fig 1:**
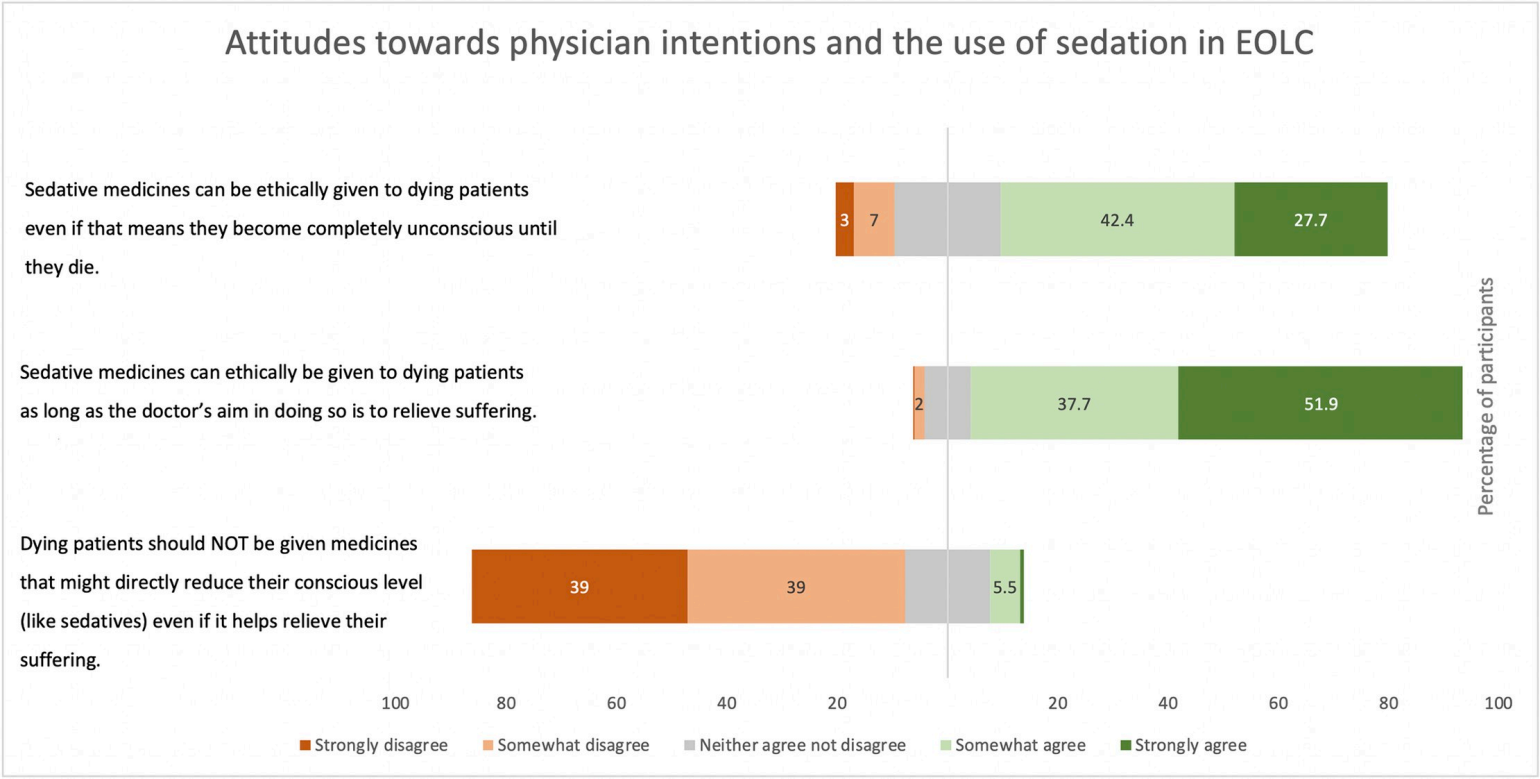
Attitudes towards physician intentions and the use of sedation (n = 509).

A majority of participants (70%) indicated that sedatives could be provided even if it makes the patient completely unconscious (Fig 1). Sixty-nine percent of respondents disagreed that doctors should never be allowed to make dying patients completely unconscious (S1 Fig); 78% disagreed that doctors should not use sedatives to alleviate suffering (Fig 1).

**Fig 2:**
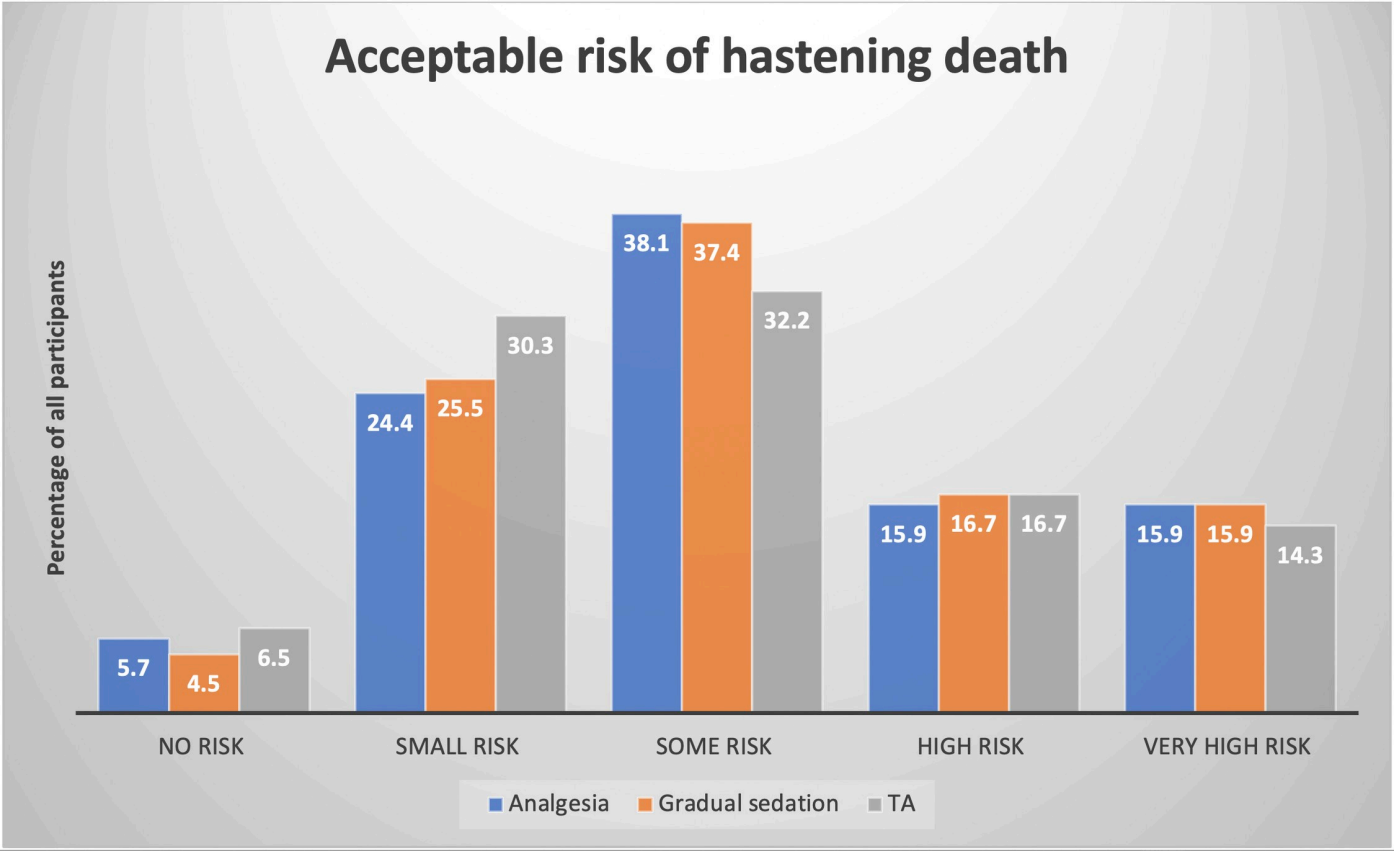
Views about the level of risk of hastening death that is acceptable when administering different end of life care options (n = 509).

When asked about the risk of hastening death with various EOLC options, the majority indicated that risk of hastening death was acceptable: more than 32% of participants indicated that ‘some’ risk of hastening death was ethically acceptable, while a further 31% believed that a high or very high risk of hastening death was acceptable (Fig 2).

Approximately, thirty-four percent of respondents indicated that anaesthetic medicines should only be available to dying patients who are expected to die within one week; a further 25% indicated it should be available for patients expected to die within twenty-four hours and 8% indicated that should be available for patients expected to die within one month. A further 27% of respondents indicated that this should be an option at any time for patients with a terminal illness, whilst 7% said anaesthetic medicines should never be an option (S2 Fig).

### Circumstances in which it is appropriate to offer TA

Approximately 80% of participants indicated that terminal anaesthesia could be ethically given to dying patients if all other options have been tried and failed. However, 51% agreed that this could ethically be provided if a dying patient requests it, even if other options have not been tried (S3 Fig).

Most survey respondents (54%) did not believe that terminal anaesthesia was equivalent to euthanasia (Table 3); however, a third regarded the two practices as equivalent.

**Table 3:** Comparing terminal anaesthesia to euthanasia (n = 509).

Statement	Response	Findings
**“Giving someone anaesthesia until death is the same as giving them a medication that ends their life.”**	Definitely yes	6.7%
	Probably yes	25.7%
	Might or might not be	13.2%
	Probably no	31.2%
	Definitely no	23.2%
**“A patient who is rendered unconscious and remains so until death is the same as a person who had their life terminated.”**	Very similar	9.6%
	Somewhat similar	27.1%
	Neither similar nor dissimilar	13.6%
	Somewhat dissimilar	30.5%
	Very dissimilar	19.1%

### Artificial nutrition and hydration at the end of life (ANH)

Participant views on providing or withholding ANH to dying patients were more divided. For a patient with one week left to live who is receiving sedation to unconsciousness, and who does not wish to receive artificial nutrition and hydration, 50% of respondents (n = 303) indicated that ANH should not be provided. For a patient who would potentially survive for four weeks, 44% percent indicated that ANH should not be provided, while 32% indicated that it should be provided (S1 Table).

Roughly, half of respondents (47%) regarded the combination of sedation to unconsciousness with withholding ANH as equivalent to intentionally ending the patient’s life (S4 Fig).

In a separate scenario, for a dying patient who has three months of life left and is refusing food and fluids and requesting sedation, 60% of participants indicated that it would be ethically acceptable for a doctor to provide sedation to unconsciousness (S5 Fig).

### Respondent characteristics and responses

There were no statistically significant differences between respondents of the two surveys when it came to the acceptability of offering titrated analgesia, gradual sedation or terminal anaesthesia to dying patients. However, respondents of the second survey had a lower acceptance rate of euthanasia than the first survey: 70.4% compared to 84.2% (p = 0.0013). The full comparison of the results of the two surveys is attached in S2 Appendix.

More females than males accepted gradual sedation (88.12% acceptance compared to 80.12%, p<0.0116). Those who accepted gradual sedation were slightly older than those who rejected it (mean age of 43.5 years compared to 32 years, p<0.0001). No statistically significant difference was found in education level between those who accepted gradual sedation and those who rejected it.

There was no statistically significant difference in age (p = 0.7689), education level (p = 0.1272) or sex (p = 0.0534) between those who accepted TA and those who rejected it. There was also no difference across those demographic parameters between those who accepted euthanasia and those who rejected it.

There was no statistically significant difference between religious and non-religious participants in accepting titrated analgesia or gradual sedation. However, religious respondents were less likely to accept TA, although a majority still endorsed this. Seventy percent of religious respondents indicated that it would be acceptable for a doctor to provide TA compared to 80% of non-religious participants (p = 0.0051).

There was also a statistically significant difference between religious and non-religious participants’ acceptance rates of euthanasia (68% of religious compared to 84% of non-religious participants, p<0.0001).

Those who rejected TA were more religious than those who accepted it (mean CRS score of 3.0 compared to 2.3, p<0.0001). Those who rejected gradual sedation and euthanasia were also slightly more religious (CRS score of 2.7 compared to 2.4 (p = 0.0128); 3.1 compared to 2.3, respectively (p<0.0001). No significant difference in religiosity was found for acceptance or rejection of titrated analgesia.

### Relationship between views on the different end of life care practices

Participants who accepted euthanasia were 8.5 times more likely to accept TA (95% CI: 4.462–16.17, p<0.0001) and 3.8 times more likely to accept gradual sedation (95% CI: 1.983–6.980, p<0.0001). Of those who accepted euthanasia, 81% accepted TA, while of respondents who rejected euthanasia, 54% accepted TA.

## Discussion

### Lay attitudes towards end-of-life care options

This is the first survey to assess the attitudes of the UK general public to sedation and anaesthesia at the end of life. In this moderately large survey, representative of the UK population for key demographic variables (The combined mean age of our two samples was 41.3 years (UK median age being 40.3 years as of 2019) [27]. The sample also predominantly identified themselves as White British (85%) (UK census data in 2011 indicates that 86% of people living in Britain identified as White) [28]. Our sample was mostly Christian (53.8%), and “no religion” was the second biggest group at 36.9%. This reflects 2011 UK data that show 59.3% of the population was Christian and 25.1% having no religious affiliation [29].), there was clear majority support (>70%) for the various end-of-life care options including analgesia, sedation, anaesthesia and euthanasia. Three-quarters of respondents indicated that it was acceptable for doctors to provide sedation with the intention of inducing unconsciousness, even if that risked hastening death.

Eighty-eight per cent wished to have the option of terminal anaesthesia available to them in their EOLC. Survey respondents were most likely to support TA where other palliative options had been tried and failed (79%). This is consistent with the views of physicians and ethicists who have justified the use of continuous and deep sedation until death as an option of last resort [10, 30, 31]. However, more than half of our survey supported TA on a dying patient’s request, even if other options have not been tried. Over half of the survey participants regarded anaesthesia in a dying patient and euthanasia as not being equivalent; a third indicated they were equivalent. Responses to questions of providing artificial nutrition and hydration to a patient receiving terminal sedation were divided.

### Comparing our findings to existing data and guidance

Our survey findings are consistent with other recent studies of public attitudes in the UK towards euthanasia. For example, 76% of our participants believed that it was ethical to allow a patient to receive euthanasia. This is in line with British Social Attitudes Surveys (BSAS), which indicate that support for euthanasia was at 77% in 2016 [32].

Although there is no existing UK data on attitudes towards sedation in EOLC, there is some data from overseas [33, 34]. A French study of 223 lay people found that 49% of participants agreed that sedation at the end of life is acceptable as long as the patient did not explicitly oppose it (this study did not specify the type of sedation) [33]. A similar Dutch study of 1,388 people found that 58% of respondents found deep sedation (to unconsciousness) acceptable [34]. This is lower than the proportion accepting rapid sedation to unconsciousness (TA) in our survey.

For titrated analgesia, a French survey of 112 lay people found that 91% accepted the use of increasing doses of morphine according to the patient’s pain levels [35]. The aforementioned Dutch study found an 82% acceptance rate for increasing morphine until the pain is under control [34]. This is similar to the 84% of respondents who supported titrated analgesia in our survey.

In our survey, a relatively large proportion of participants thought it was appropriate to render a patient unconscious until death in the patient’s final week or final 24 hours of life (34% and 25% respectively). This appears consistent with guidelines from several medical associations (e.g., Dutch and European) which limit the use of sedation to the final two weeks of life [10, 36]. However, a sizeable minority (27%) of our respondents supported TA at any time in a dying patient.

### Relationship between TA and euthanasia

We explored whether people viewed TA and euthanasia as being similar practices (implicitly testing the moral or practical comparability of the two practices). Approximately half of our participants said they were not the same (54%); a third said they were the same, and 13% were uncertain. Interestingly, this is a similar proportion to a qualitative thematic content review which analysed 37 opinion pieces (editorials, letters, comments) between 1966 and 2009. Sixty percent of opinion pieces argued that there is a moral difference between continuous deep sedation until death and physician-assisted dying; 35% argued that they are the same, and 5% had no clear position [37]. Additionally, on the status of the patient himself/herself, 36.7% of our participants shared the view previously defended in the literature that being unconscious until death is effectively the same as being dead [23], whereas 49.6% endorsed the opposing view [24] that the two states, being dead and permanently unconscious (in particular, unconsciousness that is medically induced) are not the same.

### Strengths, limitations and future studies

This was an online study representative of the UK general public across a number of demographic domains. We performed two separate surveys, drawing on different survey pools in order to increase the generalisability. The responses of our participants are broadly consistent with other UK surveys (e.g., in relation to euthanasia), and responses were consistent within the survey itself (e.g., 78.7% of participants from initial sample agreed that it was permissible to offer TA, and 78.6% later disagreed that it was “never permissible to aim at unconsciousness”). There were small differences between the surveys, which appeared to relate to the greater religiosity of the second survey cohort. However, overall, responses were remarkably similar.

In saying this, however, our sample number was modest and may not be fully representative of the wider UK population. Our scenarios were deliberately simplified. For example, our hypothetical patient was unambiguously terminally ill with one week of life left. He had the ability to make competent requests for himself without interference from family or friends. More complex, uncertain or ambiguous situations may generate different responses.

We also note that this survey was conducted in June/ July 2020 (shortly after the UK’s initial lockdown) when a substantial number of deaths related to the pandemic had been reported. It is possible that the health climate of the time influenced participant responses to be more accepting of various end-of-life care options aimed at removing suffering whilst dying.

## Ethical implications and conclusions

These survey findings cannot, in themselves, yield normative conclusions about what end of life policy ought to be. However, the views of the general public do provide valuable insights into the ethical intuitions of the community, which can be compared to ethical arguments. This data is therefore valuable for public and medical discussion and may contribute significantly to a dynamic process of reflective equilibrium.

Our survey indicates community support for euthanasia in dying patients. This support appears to be relatively stable over several decades, though assisted dying remains illegal in the UK [32]. Our survey also demonstrated support and desire for the option of rapid, deep sedation or anaesthesia at the end of life. Recent studies have indeed described the use of anaesthetic agents for patients receiving EOLC in whom other forms of palliation have not been effective [38, 39]. Relevant for public debate, TA was supported by a majority of those who supported and those who opposed euthanasia and was supported by a majority of both religious and non-religious members of the community. Neither TA nor withholding food and fluids at the end of life are illegal, though euthanasia is [26].

End of life care raises many complex ethical questions and is frequently controversial. However, two uncontroversial values endorsed by palliative care organisations and national guidelines in the UK [16] are the importance of relief of suffering and respecting patient wishes at the end of life. This study indicates that a substantial proportion of the general community support a range of options at the end of life, including some that are not currently offered in the UK.

## Supporting information

S1 Fig Views on the permissibility of aiming directly at unconsciousness in a terminally ill patient (n = 509). (PNG)

S2 Fig Views about when it would be acceptable to offer terminal anaesthesia to patients (n = 509). (PNG)

S3 Fig Terminal anaesthesia as an option of last resort (n = 509). (PNG)

S4 Fig Sedation and no artificial nutrition and hydration (ANH) is the same as euthanasia (n = 509). (PNG)

S5 Fig Voluntary stopping of eating and drinking with sedation (n = 509). (PNG)

S1 Table Should the physician provide artificial nutrition and hydration (AN)H for a patient being sedated to unconsciousness? (N = 307). (DOCX)

S1 Appendix Public attitudes towards sedation to unconsciousness in end of life care—version 2. (PDF)

S2 Appendix Separate survey analysis. (DOCX)
