# Supplementary figures and images for: British laypeople’s attitudes towards gradual sedation, sedation to unconsciousness and euthanasia at the end of life
Source: PLoS One. 2021 Mar 26;16(3):e0247193. doi: 10.1371/journal.pone.0247193 (PMC7997648; doi:10.1371/journal.pone.0247193)

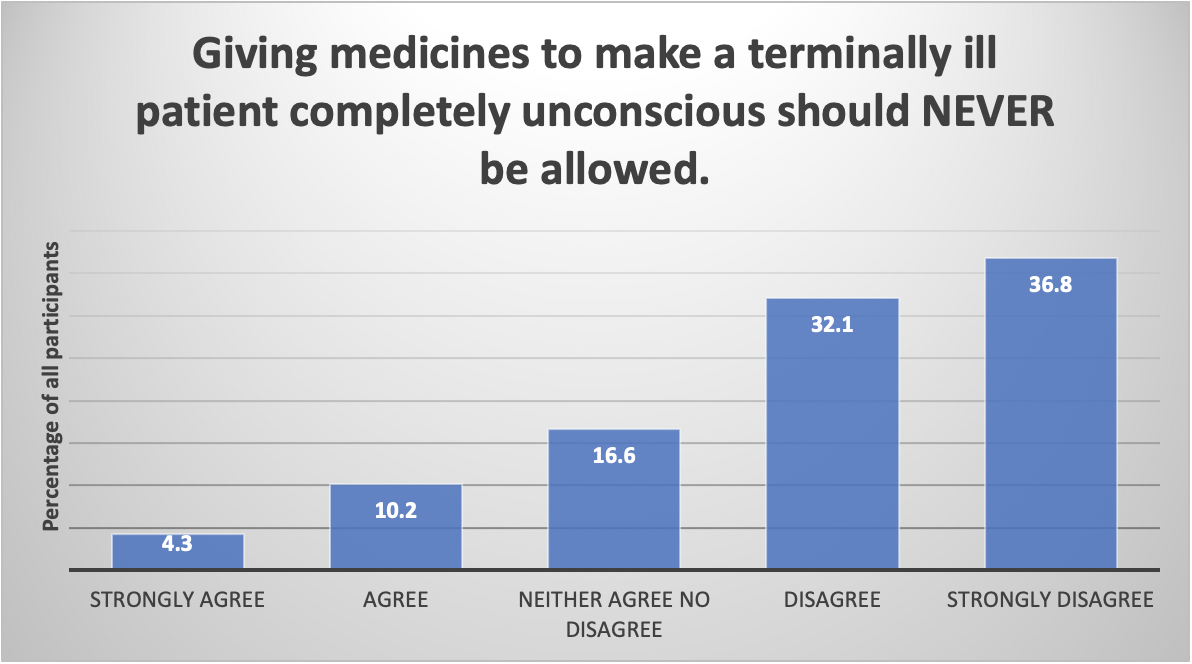

Supplement: S1 Fig — (PNG) [file pone.0247193.s001.png]

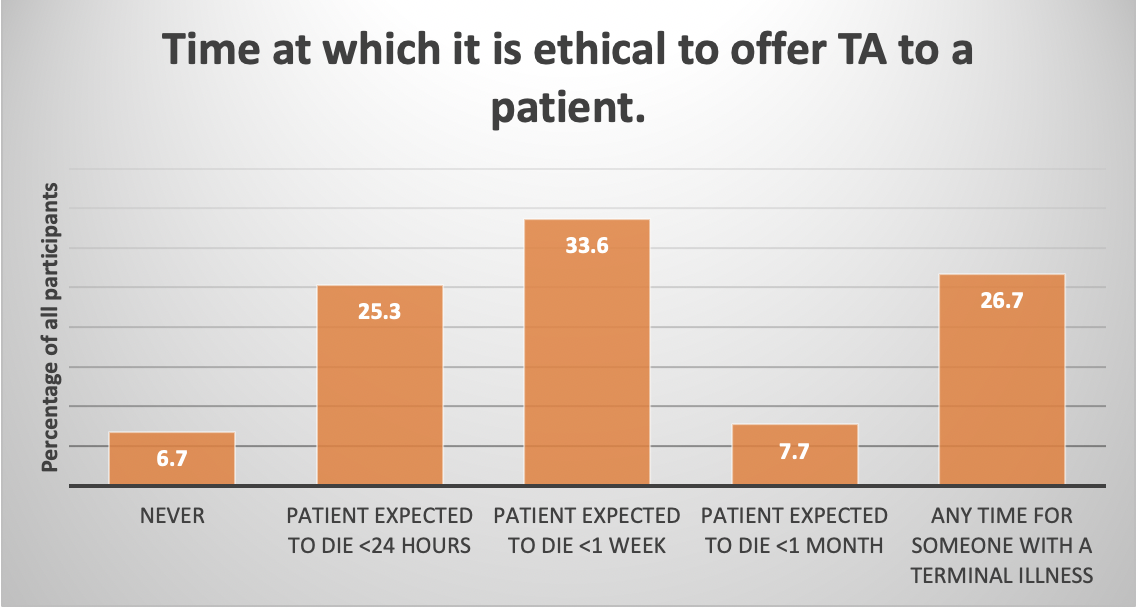

Supplement: S2 Fig — (PNG) [file pone.0247193.s002.png]

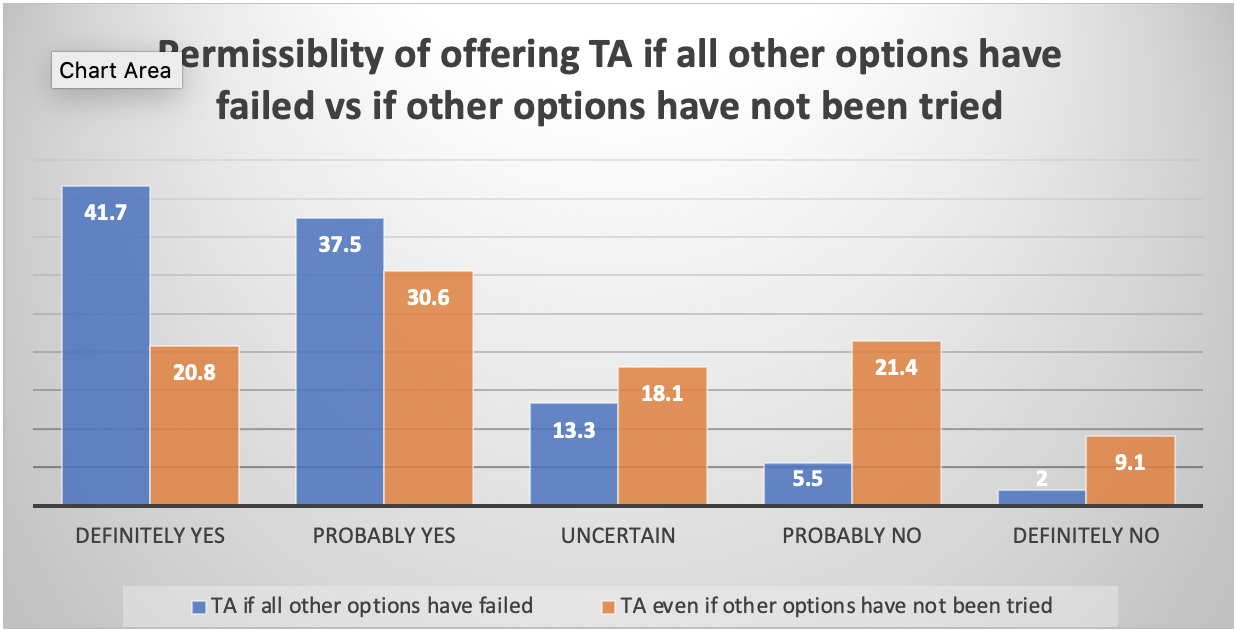

Supplement: S3 Fig — (PNG) [file pone.0247193.s003.png]

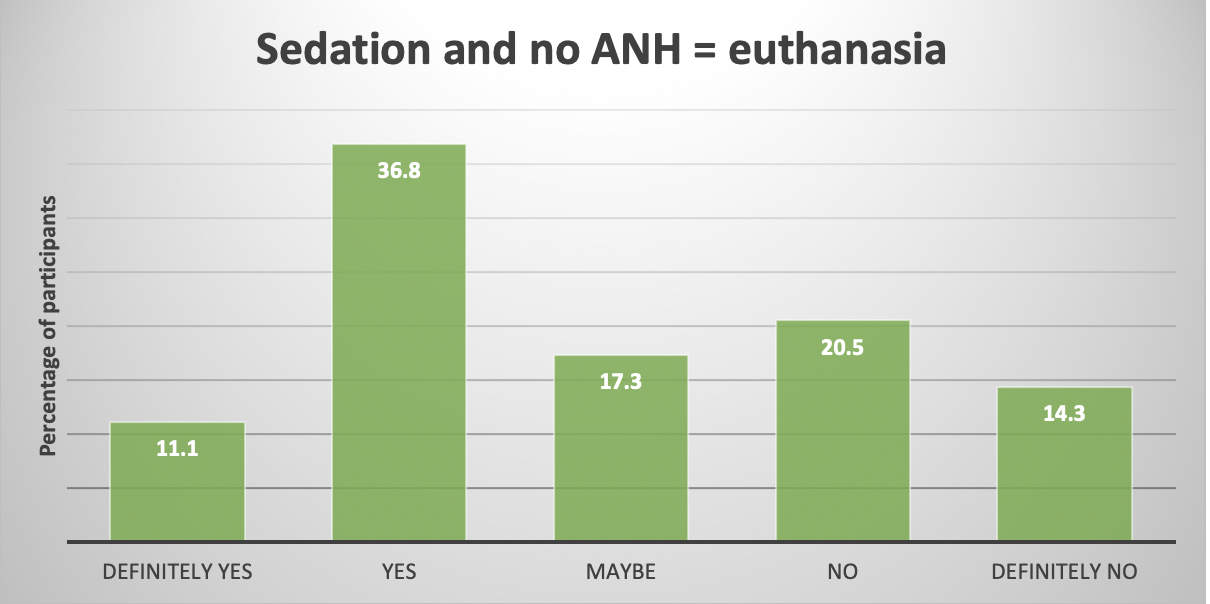

Supplement: S4 Fig — (PNG) [file pone.0247193.s004.png]

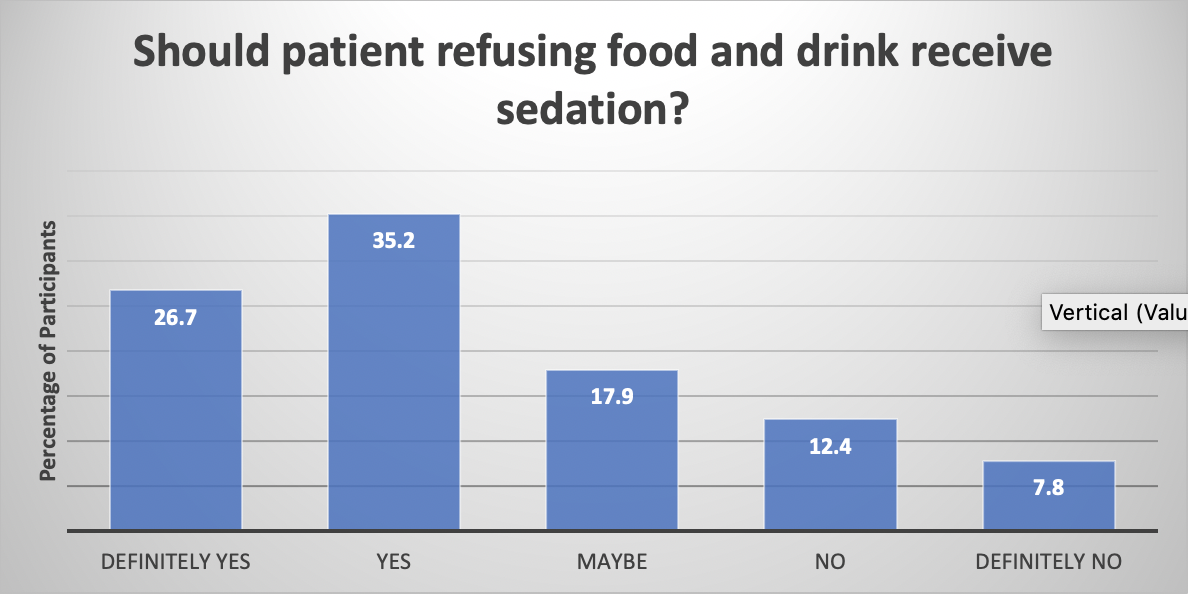

Supplement: S5 Fig — (PNG) [file pone.0247193.s005.png]
