# Supplementary material for: British laypeople’s attitudes towards gradual sedation, sedation to unconsciousness and euthanasia at the end of life
Source: PLoS One. 2021 Mar 26;16(3):e0247193. doi: 10.1371/journal.pone.0247193 (PMC7997648; doi:10.1371/journal.pone.0247193)
Supplement: S1 Table — (DOCX) [file pone.0247193.s006.docx]

**S1 Table:** Should the physician provide artificial nutrition and hydration (AN)H for a patient being sedated to unconsciousness? (N=307)

| 4 weeks of life left | | One week of life left | |
| --- | --- | --- | --- |
| Definitely yes  Yes  Maybe  No  Definitely no | 12.7%  19.2%  23.1%  31.9%  13.1% | Definitely yes  Yes  Maybe  No  Definitely no | 9.1%  20.5%  18.6%  31.9%  19.9% |
