# Supplementary material for: British laypeople’s attitudes towards gradual sedation, sedation to unconsciousness and euthanasia at the end of life
Source: PLoS One. 2021 Mar 26;16(3):e0247193. doi: 10.1371/journal.pone.0247193 (PMC7997648; doi:10.1371/journal.pone.0247193)
Supplement: S1 Appendix — (PDF) [file pone.0247193.s007.pdf]

Public Attitudes towards Sedation to Unco... 'rojects Contacts Actions Library Help 9+

Survey Actions Distributions Data & Analysis Reports

Public Attitudes towards Sedation to Unconsciousness in End of Life Care - Version 2

iQ Score: Fair Published

Introduction Block Options

Q1.1

Public attitudes towards sedation to unconsciousness in end of life care.

General information

The aim of this study is to investigate people's views on various end of life care practices and whether the use of sedation is a permissible medical treatment for dying patients. We will then use these findings from the survey and contrast them with other philosophical arguments made in the literature about the use of sedation in end of life care.

We appreciate your interest in participating in this online survey. You have been invited to participate as you are a member of the UK general public. Please read through this information carefully before agreeing to participate by ticking the 'yes' box below.

You may ask any questions before deciding to take part by contacting the researcher (details below).

The principal researcher for this project is Antony Takla (medical student) attached to the Oxford Uehiro Centre for Practical Ethics and the Department of Philosophy at the University of Oxford. This project is being completed under the supervision of Professor Julian Savulescu and Professor Dominic Wilkinson at the University of Oxford and Associate Professor Giuliana Fuscaldo at Monash University.

You will be given some medical scenarios to read, and then answer questions. It should take about 15 minutes. This is not a test of your medical knowledge, and no background knowledge is required.

Do I have to take part?

Please note that your participation is voluntary. If you do decide to take part, you may withdraw at any point during the questionnaire for any reason before submitting your answers by pressing the 'Exit' button/closing the browser. However, we are only able to reimburse participants who complete the full survey.

How will my data be used?

Your answers will be completely anonymous, and we will take all reasonable measures to ensure that they remain confidential.

Your data will be stored in a password-protected file and may be used in academic publications. Your IP address will not be stored. Research data will be stored for a minimum of three years after publication or public release.

Who will have access to my data?

Qualtrics is the data controller with respect to your personal data and, as such, will determine how your personal data is used. Please see their privacy notice here [https://www.qualtrics.com/privacy-statement/]. Qualtrics will share only fully anonymised data with the University of Oxford, for the purposes of research.

Responsible members of the University of Oxford and funders may be given access to data for monitoring and/or audit of the study to ensure we are complying with guidelines, or as otherwise required by law.

This survey is for an undergraduate research project. The project has been reviewed by, and received ethics clearance through, the University of Oxford Central University Research Ethics Committee. Reference code: R69871/RE003.

Who do I contact if I have a concern about the study or I wish to complain?

If you have a concern about any aspect of this study, please speak to Antony Takla (antony.takla@philosophy.ox.ac.uk) or Professor Dominic Wilkinson (Dominic.wilkinson@philosophy.ox.ac.uk), and we will do our best to answer your query. We will acknowledge your concern within 10 working days and give you an indication of how it will be dealt with. If you remain unhappy or wish to make a formal complaint, please contact the Chair of the Research Ethics Committee at the University of Oxford who will seek to resolve the [illegible]

matter as soon as possible:

Chair, Social Sciences & Humanities Interdivisional Research Ethics Committee; Email: ethics@socsci.ox.ac.uk; Address: Research Services, University of Oxford, Wellington Square, Oxford OX1 2JD

Content warning:

Please note that this survey is about potentially difficult end of life decisions that dying patients and their families might face. Those who have had a family member or friend recently receive end of life care may find it distressing to think about some of these decisions and may prefer not to participate. This website lists NHS-recommended helplines for support if you would like to talk to someone about your worries before or after completing this questionnaire:

https://www.nhs.uk/conditions/stress-anxiety-depression/mental-health-helplines/

Please note that you may only participate in this survey if you are at least 18 years of age.

If you agree to participate and have read the terms above, please check the relevant box below to get started.

- ○ Yes, I agree to take participate
- ○ No, I do not wish to participate

Condition: No, I do not wish to partic... Is Selected. Skip To: End of Block.

28 Jul 2020 8:16pm Andreas Kappes

Please keep all skip logic set to End of Block. We will screen people out after each block as opposed to the end of the survey. This allows us to track information on where each respondent terminates, refine our targeting, catch any potential issues, and not record the terminated response (unless you are recording partials). Thanks! - Annie

Hide Discussion | Add a Comment

Demographic Information — Block Options

Q2.1

Welcome to our study

In this study, we will ask you about your opinions on difficult decisions doctors and patients have to make. We want to know what you think is the right thing to do in these situations. The whole study will take about 15 min. Before we start, however, we want to learn a bit more about you.

Page Break

Q2.2

How old are you? (in years)

Condition: How old are you? (in years) Is Less Than 18. Skip To: End of Block.

Q2.3

How do you describe your gender?

- ◯ Male
- ◯ Female
- ◯ Other/ Prefer not to say

Q2.4

What is your ethnicity?

- ◯ White/ White British
- ◯ Asian/Asian British
- ◯ Black/ Black British
- ◯ Middle Eastern/Middle Eastern British
- ◯ Mixed/ Multiple Ethnic groups
- ◯ Other
- ◯ Prefer not to tell

Q2.5

Which part of the UK are you from?

- ◯ Northern England (North West, North East, Yorkshire and the Humber)
- ◯ Mid England (East Midlands, West Midlands & East of England)
- ◯ Southern England (South West & South East)
- ◯ Greater London
- ◯ Wales
- ◯ Scotland
- ◯ Northern Ireland

Q2.6

What is your marital status?

- ◯ Single
- ◯ Married/ Domestic partnership
- ◯ Widowed
- ◯ Divorced
- ◯ Separated
- ◯ Other

Q2.7

Do you have children? If so, how many?

No Children

Page Break

Q2.8

What is the highest level of education you have completed?

- Higher Education & professional/vocational equivalents
- A levels, vocational level 3 and equivalents
- O Level grade A*-C, vocational level 2 and equivalents
- Qualifications at level 1 and below (SATs)
- No qualifications

Q2.9

Which statement best describes your current employment status?

- Working (paid employee)
- Working (self-employed)
- Not working (temporary layoff from a job)
- Not working (looking for work)
- Not working (retired)
- Not working (disabled)
- Not working (other)
- Prefer not to answer

Q2.10

Household income:

- £19,999 or less
- £20,000 to £29,999
- £30,000 to £39,999
- £40,000 to £49,999
- £50,000 to £59,999
- £60,000 to £90,999
- £100,000 or more

Page Break

Q2.11

Please rate your own religiosity:

Not at all Religious	2	3	4	5	6	Very Religious
○	○	○	○	○	○	○

Q2.12

What is your religious affiliation?

- Christianity (e.g. Church of England, Roman Catholic, Orthodox, etc.)
- Islam
- Buddhism
- Hinduism
- Judiasm
- Non Religious (e.g. Agnostic, Atheist, No Religion)
- Other

Q2.13

How often do you think about religious issues?

- Never
- Rarely
- Occasionally
- Often
- Very Often

Q2.14

To what extent do you believe that God or something divine exists?

- Not at all
- Not very much
- Moderately
- Quite a bit
- Very much so

Q2.15

How often do you take part in religious services?

- Never
- Rarely
- Occasionally
- Often
- Very Often

Q2.16

How often do you pray?

- Never
- Rarely
- Occasionally
- Often
- Very Often

Q2.17

How often do you experience situations in which you have the feeling that God or something divine intervenes in your life?

- Never
- Rarely
- Occasionally
- Often
- Very Often

Page Break

Q2.18

We care about the quality of our data. In order for us to get the most accurate measures of your opinions, it is important that you thoughtfully provide your best answers to each question in this survey.

Page Break

Pain Killer Scenario

Block Options

Q3.1

Ethical Medical Treatment

Now, we are interested in hearing your views on what you consider ethical medical treatment. This is **NOT** an assessment of your understanding of what the law and medical guidelines currently allow. Tell us whatever you think is the right thing to do in each of the described situations.

Page Break

Q3.2

On the following pages, we will describe the case of Mr. Thomson, a person with end-stage cancer. He will die soon. Please read the descriptions carefully and then answer the questions according to your beliefs and opinions.

Page Break

Q3.3

Mr. Thomson is a 75-year-old man with end-stage cancer. He has approximately one week of life left. Mr. Thomson is admitted to his local hospital because of very strong pain. He has no family or friends to be with him at this time.

Page Break

Q3.4

When answering each of the following questions, always assume that Mr. Thomson has consented to the treatment; nothing is done against his will.

Page Break

Q3.5

The doctor sees Mr. Thomson and offers him a low dose of painkillers that he thinks will help Mr. Thomson. Because it's a low dose of the medication, Mr. Thomson is not likely to suffer side effects (like reduced consciousness) or die sooner than if he had not received the medication at all. Do you think this is an ethical treatment option to offer to Mr. Thomson?

- Definitely yes
- Probably yes
- Might be or might not be
- Probably not
- Definitely not

Page Break

Q3.6

If Mr. Thomson's pain does **not** go away after the small dose of pain killers, the doctor can gradually increase the dose until Mr. Thomson's pain goes away. Note that as you increase the dose, the risk of side effects (like reduced consciousness) increases and it is slightly more likely that Mr. Thomson may die sooner than if he had not received the medications at all. Do you think this is an ethical treatment option to offer to Mr. Thomson?

- Definitely yes
- Probably yes
- Might be or might not be
- Probably not
- Definitely not

Page Break

Q3.7

The previous question was testing whether you thought gradually increasing pain killers is an ethical treatment option to offer to patients in general. Now we want to know what you would choose for yourself or a close family member.

Page Break

Q3.8

If you, or a family member, were in Mr. Thomson's position, would you request that pain killers be gradually increased until you are no longer in pain (despite increased risk of hastening death or reducing consciousness)?

- Extremely likely
- Somewhat likely
- Neither likely nor unlikely
- Somewhat unlikely
- Extremely unlikely

Page Break

Gradual Sedation Scenario Block Options

Q4.1

Mr. Thomson initially responds well to the increasing doses of pain killers. However, after two days, his pain returns again and is no longer responding to the pain killers.

Page Break

Q4.2

The doctor sees Mr. Thomson and offers to add a small dose of **a sedative** alongside the pain killers to help relieve some of his suffering by reducing his level of consciousness. There is a small risk that this sedative will make Mr. Thomson die sooner than if he did not take it. Do you think this is an ethical treatment option to offer to him?

- Definitely yes
- Probably yes
- Might or might not
- Probably not
- Definitely not

Page Break

Q4.3

If the small dose of the sedative did not work, the doctor can keep increasing the dose until Mr. Thomson seems to be not suffering. Note that there is a chance that Mr. Thomson might become completely unconscious and die sooner than if he did not receive the larger dose of the sedative. Is this an ethical treatment option to offer to him?

- Definitely yes
- Probably yes
- Might or might not
- Probably not
- Definitely not

Page Break

Q4.4

The previous question was about what you thought was ethical treatment to offer to patients in general. Now we want to know what you would choose for yourself or a close family member.

Page Break

Q4.5

If you, or a family member, were in Mr. Thomson's position, would you request that sedatives be increased gradually until you are no longer in pain (despite increased risk of becoming unconscious and dying sooner)?

- Extremely likely
- Somewhat likely
- Neither likely nor unlikely
- Somewhat unlikely
- Extremely unlikely

Page Break

Anaesthesia Scenario — Block Options

Q5.1

Mr. Thomson says that he does not want to take any risk of still being in pain with the gradual increase of sedatives and instead wants to be made completely unconscious and pain free straight away.

The doctor offers to provide **anaesthetic medicines** (like those given during surgery) which will make him completely unconscious immediately. There is a small risk that this anaesthetic medicine will make Mr. Thomson die sooner than if he did not take it. Do you think this is an ethical treatment option to offer to him?

- Definitely yes
- Probably yes
- Might or might not
- Probably not
- Definitely not

Page Break

Q5.2

The previous question was testing whether you thought making a patient completely unconscious is an ethical treatment option to offer. Now we want to know what you would choose for yourself or a close family member.

Page Break

Q5.3

If you, or a family member, were in Mr. Thomson's position, would you request to receive an anaesthetic medicine that would make you completely unconscious?

- Extremely likely
- Somewhat likely
- Neither likely nor unlikely
- Somewhat unlikely
- Extremely unlikely

Q5.4

Whether you chose to take up this option of anaesthesia or not in the previous question, would you still like to have that option available to you anyway?

- Definitely yes
- Probably yes
- Might or might not
- Probably not
- Definitely not

Q5.5

Mr. Thomson says that he understands the risks of anaesthesia (being made unconscious until death) and wants to receive that option anyway. If you are reading this text please choose the Definitely not option for this question.

Do you think this is an ethical treatment option?

- Definitely yes
- Probably yes
- Might or might not
- Probably not
- Definitely not

Condition: Definitely not Is Not Selected. Skip To: End of Block.

Page Break

Attitudes towards Euthanasia Scenario — Block Options

Q6.1

Now imagine that instead of the treatment options mentioned earlier, Mr. Thomson expresses that he wants to die immediately.

Page Break

Q6.2

Do you think Mr. Thomson should have the option of receiving a medicine that would end his life if he wishes to?

- Definitely yes
- Probably yes
- Might or might not
- Probably not
- Definitely not

Page Break

Q6.3

If you, or a family member, were in Mr. Thomson's position, would you request to have your life ended by the physician?

- Definitely yes
- Probably yes
- Might or might not
- Probably not
- Definitely not

Q6.4

Whether you would take up this option of having a physician end your life or not, would you still like to have that option available to you anyway?

- Definitely yes
- Probably yes
- Might or might not
- Probably not
- Definitely not

Page Break

General attitudes towards pain relieving medicines Block Options

Q7.1

We are now done with scenario based questions. In the following section, we want to learn more about your general attitudes towards **pain killers** and how you feel about the different risks that may come with giving these medicines.

Page Break

Q7.2

If pain killers are given to dying patients, there can be a risk that they would die sooner than they would otherwise.

How high a risk of death happening sooner would be ethically acceptable?

(Assume the patient has agreed to this, and assume that the doctor's aim is to relieve pain).

- No risk of speeding up death is acceptable
- Small risk of speeding up death is acceptable
- Some risk of speeding up death is acceptable
- High risk of speeding up death is acceptable
- Very high risk of speeding up death is acceptable

Page Break

Q7.3

Do you agree or disagree with the following statements?

Q7.4

Pain killers can be given to dying patients as long as the doctor's aim in doing so is to relieve pain not speed up death.

- Strongly agree
- Somewhat agree
- Neither agree nor disagree
- Somewhat disagree
- Strongly disagree

Q7.5

Pain killers can be given to dying patients even if the doctor's aim is to hasten a patient's death.

- Strongly agree
- Somewhat agree
- Neither agree nor disagree
- Somewhat disagree
- Strongly disagree

Page Break

General attitudes towards Sedative medicines

Block Options

Q8.1

In the following set of questions, we want to learn more about your general attitudes towards **sedatives** and how you feel about the different risks that may come with giving these medicines.

Q8.2

Do you agree or disagree with the following statements?

Q8.3

Sedative medicines can be ethically given to dying patients even if that means they become completely unconscious until they die.

- Strongly agree
- Somewhat agree
- Neither agree nor disagree
- Somewhat disagree
- Strongly disagree

Q8.4

Sedative medicines can ethically be given to dying patients as long as the doctor’s aim in doing so is to relieve suffering.

- Strongly agree
- Somewhat agree
- Neither agree nor disagree
- Somewhat disagree
- Strongly disagree

Page Break

Q8.5

Dying patients should **NOT** be given medicines that might directly reduce their conscious level (like sedatives) even if it helps relieve their suffering.

- Strongly agree
- Somewhat agree
- Neither agree nor disagree
- Somewhat disagree
- Strongly disagree

Q8.6

If sedative medicines are given to dying patients, there can be a risk that they would die sooner than they would otherwise.

How high a risk of death happening sooner would be ethically acceptable?

(Assume the patient has agreed to this, and assume that the doctor's aim is to relieve suffering).

- No risk of speeding up death is acceptable
- Small risk of speeding up death is acceptable
- Some risk of speeding up death is acceptable
- High risk of speeding up death is acceptable
- Very high risk of speeding up death is acceptable

Q8.7

If sedative medicines are given to dying patients, there can be a risk that the patient is still suffering but not able to communicate that suffering because they are too drowsy. What level of risk of that happening would be ethically acceptable?

- No risk at all
- Small risk
- Some risk
- High risk
- Very high risk

Attitudes towards Anaesthetic medicines Block Options

Q9.1

In the following set of questions, we want to learn more about your general attitudes towards **anaesthetic medicines** (making the patient completely unconscious straight away) and how you feel about the different risks that may come with giving this type of medicine.

Q9.2

Anaesthetic medicines (making a patient completely unconscious) can be ethically given to dying patients if all other options to make them comfortable have been tried and failed.

- Strongly agree
- Somewhat agree
- Neither agree nor disagree
- Somewhat disagree
- Strongly disagree

Q9.3

Anaesthetic medicines (making a patient completely unconscious) can be ethically given to dying patients even if other options to make them comfortable have not yet been tried because that's what the patient wants.

- Strongly agree
- Somewhat agree
- Neither agree nor disagree
- Somewhat disagree
- Strongly disagree

Q9.4

Anaesthetic medicines (making a patient completely unconscious) can be ethically given to dying patients as long as the patient has consented to this.

- Strongly agree
- Somewhat agree
- Neither agree nor disagree
- Somewhat disagree
- Strongly disagree

Q9.5

Anaesthetic medicines (making a patient completely unconscious) can be ethically given to dying patients if the patient appears to be suffering even if they aren't able to consent to this.

- Strongly agree
- Somewhat agree
- Neither agree nor disagree
- Somewhat disagree
- Strongly disagree

Q9.6

Giving strong sedative medicines to make a dying patient completely unconscious should **never** be allowed.

- Strongly agree
- Somewhat agree
- Neither agree nor disagree
- Somewhat disagree
- Strongly disagree

Q9.7

If anaesthetic medicines are given to dying patients, there can be a risk that they would have an adverse reaction to the medication. If you are reading this, please choose the Small risk option.

How high a risk of an adverse reaction happening would be ethically acceptable?

- No risk
- Small risk
- Some risk
- High risk
- Very high risk

Condition: Small risk Is Not Selected. Skip To: End of Block.

Q9.8

If anaesthetic medicines are given to dying patients, there can be a risk that they would die sooner than they would otherwise.

How high a risk of death happening sooner would be ethically acceptable?

(Assume the patient has agreed to this, and assume that the doctor's aim is to relieve pain).

- No risk of speeding up death is acceptable
- Small risk of speeding up death is acceptable
- Some risk of speeding up death is acceptable
- High risk of speeding up death is acceptable
- Very high risk of speeding up death is acceptable

Page Break

Timing of Anaesthesia   Block Options

Q10.1

When should anaesthetic medicines be offered as an option for dying patients who wish to be made unconscious until they die?

- Never
- Only in patients expected to die in next 24 hours or less
- In patients expected to die within a week
- In patients expected to die within a month
- In any patient with a terminal illness

Page Break

Q10.2

Dying patients who receive sedation are not able to eat and drink normally. In those circumstances food and fluids can be delivered artificially via a small tube inserted into their stomach through their nose.

However, sometimes the patient does not wish to receive artificial food and fluids if they are not going to survive.

Page Break

Q10.3

In all of the following scenarios, the patient has asked to receive sedation to unconsciousness and indicated that they do **not** wish to receive artificial food and fluids.

Page Break

Q10.4

A terminally ill patient asks to receive sedation and they have 4 weeks of life left.

If they receive artificial food and fluids they will potentially remain alive for 4 weeks (but unconscious).

If they do not receive food and fluids through an artificial tube, they would probably die sooner (for example in 1-2 weeks), but they will not experience suffering from hunger or thirst because they are unconscious.

Do you think the doctor should administer food and fluids artificially in this case?

- Definitely yes
- Probably yes
- Might or might not
- Probably not
- Definitely not

Q10.5

Another patient has only one week of life left.

If they receive artificial food and fluids they will potentially remain alive for 1 week (but unconscious).

If they do not receive food and fluids through an artificial tube, this is not likely to make a difference to their life expectancy. They will not experience suffering from hunger or thirst (because they are unconscious).

Do you think the doctor should administer food and fluids artificially in this case?

- Definitely yes
- Probably yes
- Might or might not
- Probably not
- Definitely not

Q10.6

Another patient also has a terminal illness and they have 3 months of life left. They are being provided with palliative care, but their pain is not completely controlled. The patient expresses that they do not wish to continue living.

The patient decides to stop eating and drinking by mouth, and refuse any form of artificial food and fluids. They ask their doctor to sedate them so that they are unconscious and do not suffer from pain, hunger or thirst as they die in the coming 1-2 weeks.

Do you think it is ethical for the doctor to provide this patient with the sedation they requested?

- Definitely yes
- Probably yes
- Might or might not
- Probably not
- Definitely not

Page Break

Anaesthesia vs Euthanasia — Block Options

Q11.1

This section aims to assess whether you view making someone unconscious until death, and ending their life as similar or different.

Q11.2

Do you think that giving someone a medication to make them unconscious until death is the same as giving them a medication that ends their life immediately?

- Definitely yes
- Probably yes
- Might or might not
- Probably not
- Definitely not

Q11.3

Do you think that an unconscious patient who remains so until death is the same as a person who had their life terminated?

- Very similar
- Somewhat similar
- Neither similar nor dissimilar
- Somewhat dissimilar
- Very dissimilar

Q11.4

Do you think that giving someone a medication to sedate them, and also **NOT** giving them artificial food and fluids is the same as giving them a medication that ends their life?

- Definitely yes
- Probably yes
- Might or might not
- Probably not
- Definitely not

What do you want in your end of life care? — Block Options

Q12.1

Finally, please select all the options that you would like to have available to you for your end of life care. Note, this is not asking which options you would choose, but which one's you'd like to have there for you in case you want to choose them.

- Pain killers
- Gradual sedation
- Anaesthetic medicines (immediate unconsciousness)
- Euthanasia (having your life terminated by the physician)

Add Block

End of Survey — Survey Termination Options...

CITY UNIVERSITY OF LONDON EST 1894

Qualtrics.com Contact Information Legal
