# Supplementary material for: British laypeople’s attitudes towards gradual sedation, sedation to unconsciousness and euthanasia at the end of life
Source: PLoS One. 2021 Mar 26;16(3):e0247193. doi: 10.1371/journal.pone.0247193 (PMC7997648; doi:10.1371/journal.pone.0247193)
Supplement: S2 Appendix — (DOCX) [file pone.0247193.s008.docx]

## S2 Appendix – Separate Survey Analysis

There were no significant differences between the two survey cohorts to key questions regarding terminal anaesthesia (such as the permissibility of offering it to patients and proportion of participants wanting to have that option available for their end of life care). There was also no difference in response across the two groups regarding the equivalence of TA and euthanasia.

There were small differences between the survey cohorts in regard to the acceptable timing of offering TA and acceptable risk of hastening death. For example, 21% of participants from the first survey compared to 28% from the second survey said it was ethical to offer TA to patients expected to die in 24 hours; and 35.2% compared to 32.6% respectively for patients expected to die within one week. Below are the separate results from the first and second surveys.

## Key findings

Table 1: Acceptance rates of different end of life care options (separate responses: initial n=202, replication n=307).

|  | | Ethical to offer patients. | | Participant would choose it for themselves or family member. | | Participant would like to have the option available to them regardless. | | |
| --- | --- | --- | --- | --- | --- | --- | --- | --- |
|  |  | Initial | Replication | Initial | Replication | Initial | Replication | |
| Gradual sedation | Yes  Uncertain  No | 71.8%  12.9%  15.3% | 72.6%  17.3%  10.1% | 80.7%  8.2%  10.9% | 79.8%  11.1%  9.1% | Not assessed. ^[[1]](#footnote-1)^ | | |
| Terminal Anaesthesia | Yes  Uncertain  No | 78.7%  13.9%  7.4% | 71.7%  15.3%  13.0% | 69.3%  11.9%  18.8% | 61.6%  19.5%  18.9% | 89.1%  5.5%  5.4% | | 88.0%  6.8%  5.2% |
| Euthanasia | Yes  Uncertain  No | 84.2%  3.5%  12.3% | 70.4%  11.1%  18.5% | 53%  27.7%  19.3% | 50.5%  22.5%  27.0% | 83.2%  6.4%  10.4% | | 76.2%  6.8%  17.0% |

Figure 1: When should Terminal Anaesthesia be made available to patients? (Initial n=202, replication n=307).

Figure 2: Terminal Anaesthesia when all other options have failed versus when other options have not been tried. (Initial n=202, replication n=307).

Figure 3: Acceptable risk of hastening death when administering Terminal Anaesthesia (initial n=202, replication n=307).

Figure 4: Permissibility of aiming at unconsciousness (initial n=202, replication n=307).

Table 2: Comparing anaesthesia to euthanasia.

| Question | Response | Initial Study (n=202) | Replication Study (n=307) |
| --- | --- | --- | --- |
| “Giving someone anaesthesia until death is the same as giving them a medication that ends their life” | Yes  Uncertain  No | 33.7%  12.9%  53.4% | 31.6%  13.4%  55.0% |
| “An unconscious patient who remains so until death is the same as a person who had their life terminated” | Yes  Uncertain  No | 38.6%  10.9%  50.5% | 35.5%  15.3%  49.2% |

1. This practice is also already available to patients receiving EOLC. [↑](#footnote-ref-1)
